# Small Cell Neuroendocrine Carcinoma of the Gallbladder: Natural History and Therapeutic Challenges

**DOI:** 10.7759/cureus.93358

**Published:** 2025-09-27

**Authors:** Niket Shah, Alicia Edwards, Jason P Law, Nasreen Al-Qadi, Mukta Sharma

**Affiliations:** 1 Internal Medicine, Michigan State University College of Human Medicine, Lansing, USA; 2 General Surgery, Michigan State University College of Human Medicine, Lansing, USA

**Keywords:** biliary tract malignancy, duodenal mass obstruction, gallbladder neuroendocrine carcinoma, gastric pneumatosis, inferior vena cava invasion, ki-67 proliferation index, platinum etoposide chemotherapy, porta hepatis lymphadenopathy, small cell carcinoma, unresectable gallbladder cancer

## Abstract

Small cell neuroendocrine carcinoma (SNEC) of the gallbladder is an extremely rare and aggressive primary gallbladder malignancy with poor prognosis. A 65-year-old woman presented with constipation followed by jaundice and right upper quadrant pain one month later. Imaging revealed a porta hepatis mass with hepatic and duodenal involvement. Fine needle aspiration confirmed metastatic small cell carcinoma positive for synaptophysin, chromogranin, and CDX2 with Ki-67 >70%. Despite planned platinum-etoposide chemotherapy, rapid disease progression with inferior vena cava invasion precluded treatment. The patient died 13 weeks after symptom onset. This case demonstrates the aggressive natural history of gallbladder SNEC. Early tissue diagnosis and prompt platinum-etoposide chemotherapy initiation while performance status permits remain critical for meaningful survival benefit.

## Introduction

Gallbladder cancer carries a poor prognosis with five-year overall survival less than 20% [1]. While adenocarcinoma comprises over 83% of cases, small cell neuroendocrine carcinoma accounts for only 0.5% of gallbladder malignancies [2]. Unlike neuroendocrine tumors at other gastrointestinal sites, gallbladder neuroendocrine carcinomas demonstrate particularly aggressive behavior [1]. The rarity of this entity limits understanding of optimal management. Median survival for unresectable disease is 2-4 months without treatment [2]. We present a case illustrating the rapid progression and therapeutic challenges of this malignancy.

## Case presentation

**Figure 1:**
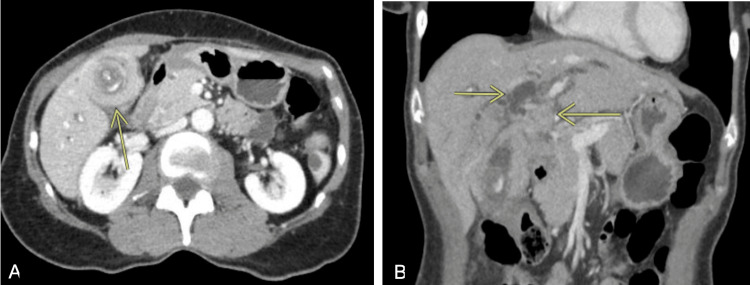
Initial CT demonstrating a large mass in the fundus of the gallbladder (A) with involvement of the liver, porta hepatis and intraductal biliary dilation (B).

A 65‑year‑old woman with no significant past medical history initially presented to her primary care physician with intermittent abdominal pressure and constipation. Baseline laboratory studies were unremarkable, and she was managed conservatively. One month later, she developed jaundice and right upper‑quadrant pain. Physical examination revealed an ill‑appearing, jaundiced woman with hepatomegaly. Admission laboratory evaluation demonstrated mixed hepatocellular‑cholestatic injury and mild hypoalbuminemia (Table 1). Initial computed tomography demonstrated a large porta hepatis mass involving the gallbladder, liver, and hepatic flexure (Figure 1).

**Table 1:** Admission laboratory findings (jaundice presentation) Laboratory-specific reference range

Test (units)	Result	Reference Range
WBC (×10³/µL)	8.3	4.0 – 10.0
Hemoglobin (g/dL)	11.7 (Low)	12.0 – 16.0
Platelets (×10³/µL)	297	150 – 450
Sodium (mmol/L)	133 (Low)	135 – 146
Potassium (mmol/L)	3.9	3.5 – 5.1
Chloride (mmol/L)	100	98 – 108
Bicarbonate (mmol/L)	27	21 – 31
BUN (mg/dL)	9	8 – 20
Creatinine (mg/dL)	0.46 (Low)	0.50 – 1.00
Glucose, Random (mg/dL)	154	70 – 180
Total Protein (g/dL)	5.8 (Low)	6.0 – 8.3
Albumin (g/dL)	3.0 (Low)	3.5 – 4.9
AST (U/L)	82 (High)	10 – 49
ALT (U/L)	91 (High)	10 – 49
Alkaline Phosphatase (U/L)	616 (High)	40 – 116
Total Bilirubin (mg/dL)	1.9 (High)	0.2 – 1.2
Lipase (U/L)	96 (High)	12 – 53
Magnesium (mg/dL)	1.7	1.6 – 2.6
TSH (µIU/mL)	2.19	0.30 – 5.0

**Figure 2:**
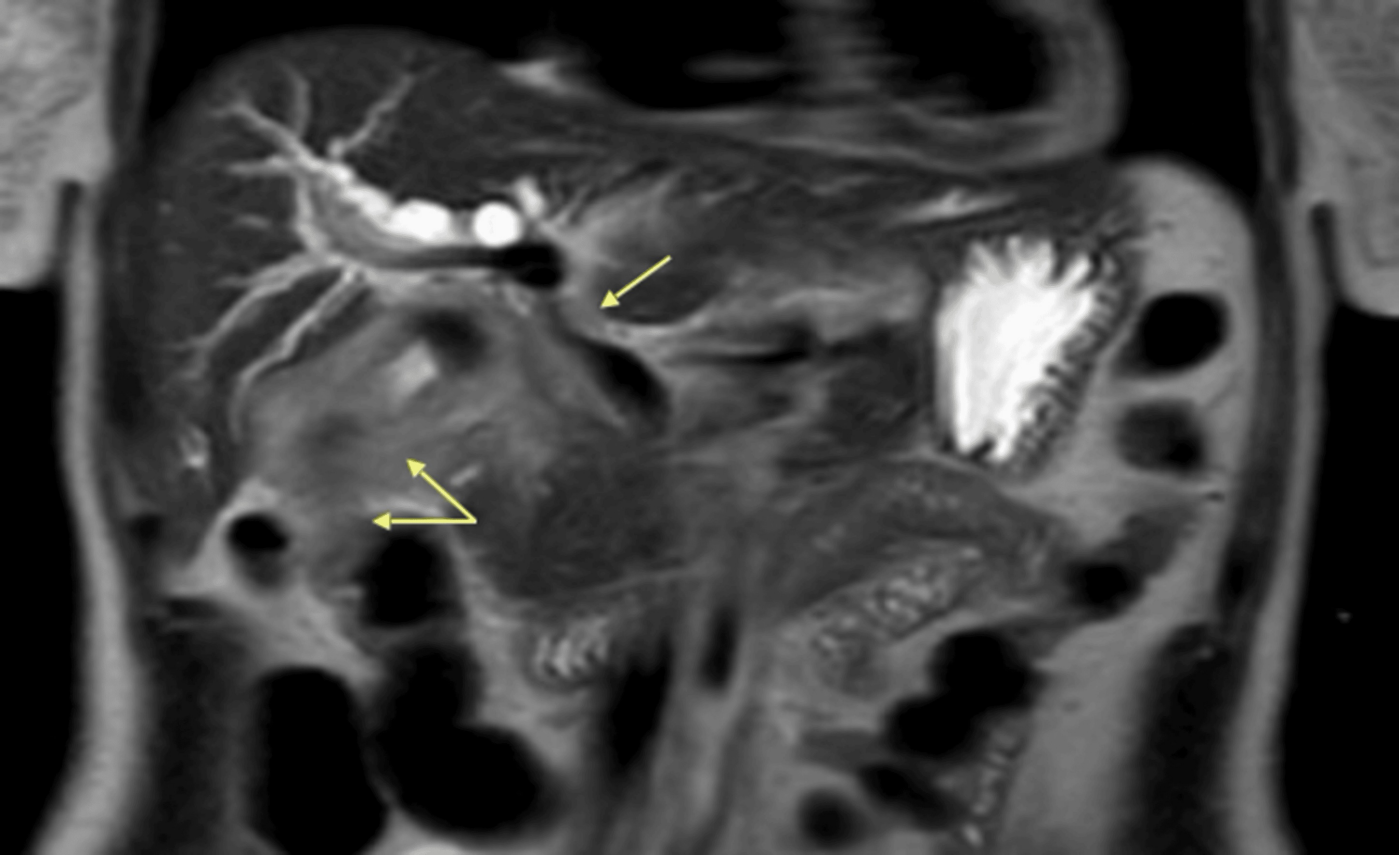
MRCP demonstrating large gallbladder mass with hepatic flexure involvement and encasement of the right portal vein (yellow arrows) MRCP: Magnetic Resonance Cholangiopancreatography

Magnetic resonance cholangiopancreatography demonstrated locally aggressive disease with hepatic flexure involvement and right portal vein encasement, classified as cT4N1 (Figure 2).

**Figure 3:**
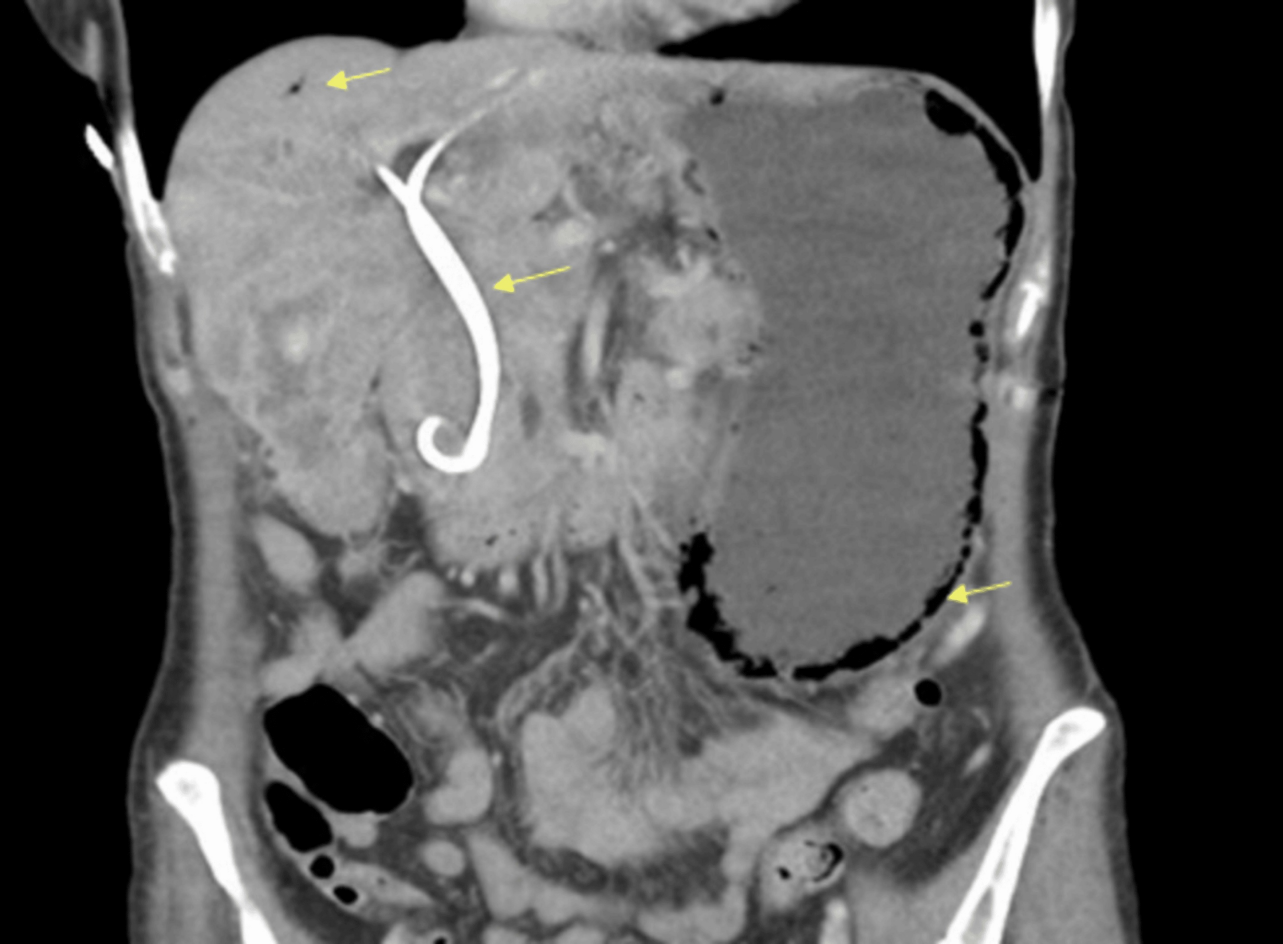
CT AP demonstrating significant gastric pneumatosis, a biliary stent and some portal venous gas in the setting of the known gallbladder small cell carcinoma

Endoscopic retrograde cholangiopancreatography revealed a 3-cm common bile duct stricture requiring stent placement. Endoscopic ultrasound-guided fine needle aspiration of porta hepatis lymph nodes yielded metastatic carcinoma consistent with small cell type. Immunohistochemical analysis demonstrated positivity for pan-cytokeratin, cytokeratin 7, synaptophysin, chromogranin A, CDX2, and CD56. Ki-67 proliferation index exceeded 70%. TTF-1 and cytokeratin 20 were negative, excluding pulmonary origin. Positron emission tomography confirmed a hypermetabolic gallbladder mass extending to hepatic segments 4B and 5 with regional lymphadenopathy. Before chemotherapy initiation, the patient developed intractable nausea and vomiting. Repeat imaging showed extensive gastric pneumatosis with portal venous gas in the setting of the known gallbladder small cell carcinoma (Figure 3).

**Figure 4:**
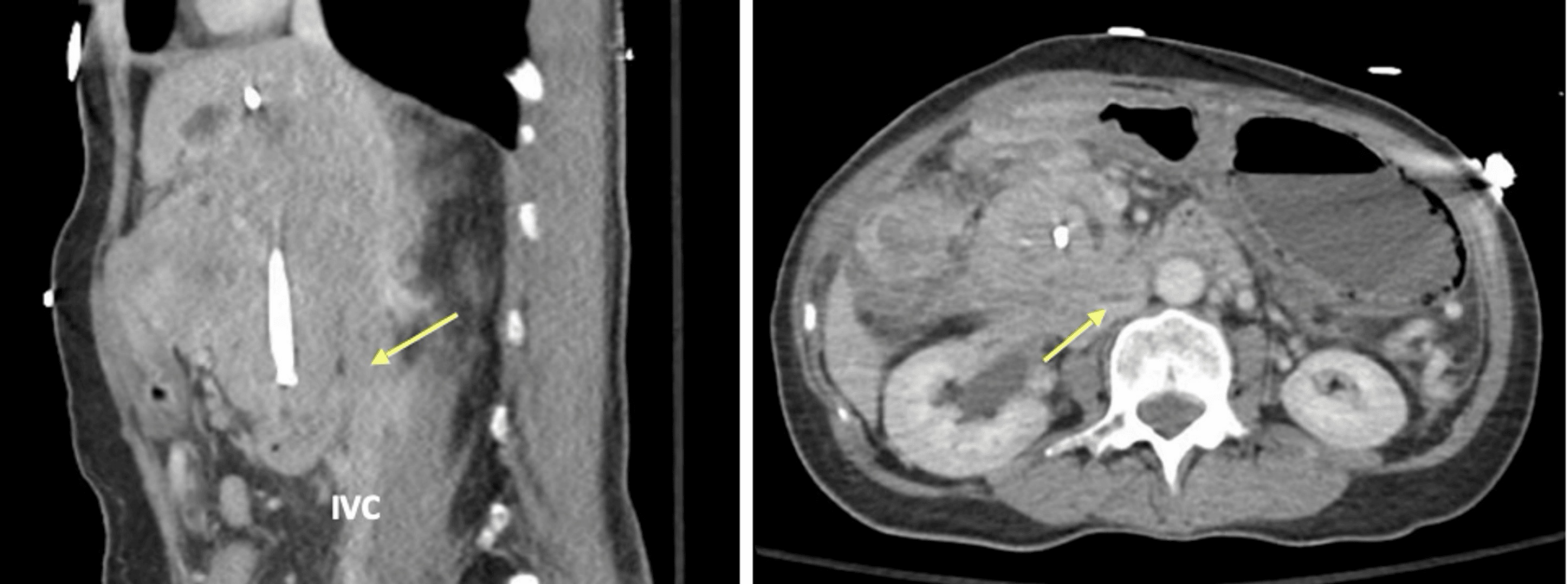
Sagittal (A) and Axial (B) views of CT AP demonstrating IVC invasion (yellow arrows) from the rapidly expanding gallbladder small cell carcinoma IVC: Inferior Vena Cava

Upper endoscopy revealed hemorrhagic gastritis and a large ulcerated duodenal mass. Biopsy confirmed high-grade neuroendocrine carcinoma morphologically identical to the primary tumor. Further imaging demonstrated rapid disease progression with inferior vena cava invasion, precluding systemic therapy (Figure 4). 

The patient transitioned to hospice care and died 13 weeks after initial symptom onset.

## Discussion

This case exemplifies the aggressive biology of gallbladder SNEC. The 13-week survival from symptom onset to death aligns with historical data for untreated disease [2]. Initial nonspecific symptoms delayed diagnosis by one month, during which significant disease progression occurred. Platinum-etoposide chemotherapy is the standard first-line treatment for SNEC, though the benefit requires adequate performance status [2]. Our patient's rapid clinical deterioration with vascular invasion precluded treatment, highlighting the narrow therapeutic window. Literature suggests that patients with performance status 0-2 benefit from systemic therapy, while those with status 3-4 typically receive supportive care.

Previous studies confirm that gallbladder neuroendocrine carcinomas typically present at advanced stages. Among 21 patients in one series, 85.8% presented with abdominal pain and 28.6% with jaundice [3]. The immunohistochemical profile proved diagnostic: Chromogranin A and synaptophysin show positivity rates of 81-86% in gallbladder neuroendocrine carcinomas [3]. The high Ki-67 index (>70%) indicated poorly differentiated disease, consistent with published data showing most cases exceed 20% [4]. The pathogenesis likely involves gallbladder epithelial metaplasia from chronic inflammation [4]. Our patient's ultrasound one month prior showed gallbladder wall thickening with stones, supporting this hypothesis. While gallbladder neuroendocrine carcinomas show worse survival than adenocarcinomas in unmatched cohorts, stage-matched analysis reveals similar outcomes [4]. This suggests that late diagnosis rather than inherent biology drives poor outcomes.

## Conclusions

SNEC of the gallbladder remains a therapeutic challenge. This case demonstrates a typical presentation with nonspecific symptoms, rapid progression, and death within months despite planned treatment. Early tissue diagnosis through aggressive evaluation of gallbladder masses and prompt chemotherapy initiation, while performance status permits, may offer an opportunity for modest survival extension, though prognosis remains poor even with treatment, given the aggressive biology of this malignancy.
